# Administration of CORM-2 inhibits diabetic neuropathy but does not reduce dyslipidemia in diabetic mice

**DOI:** 10.1371/journal.pone.0204841

**Published:** 2018-10-04

**Authors:** Karen Alejandra Méndez-Lara, David Santos, Núria Farré, Sheila Ruiz-Nogales, Sergi Leánez, José Luis Sánchez-Quesada, Edgar Zapico, Enrique Lerma, Joan Carles Escolà-Gil, Francisco Blanco-Vaca, Jesús María Martín-Campos, Josep Julve, Olga Pol

**Affiliations:** 1 Grup de Bases Metabòliques de Risc Cardiovascular, Institut de Recerca de l’Hospital de la Santa Creu i Sant Pau & Institut d’Investigació Biomèdica Sant Pau (IIB Sant Pau), Barcelona, Spain; 2 Departament de Bioquímica i Biologia Molecular, Universitat Autònoma de Barcelona, Barcelona, Spain; 3 CIBER de Diabetes y Enfermedades Metabólicas Asociadas, CIBERDEM, Barcelona, Spain; 4 Grup de Neurofarmacologia Molecular, Institut de Recerca de l’Hospital de la Santa Creu i Sant Pau & Institut d’Investigació Biomèdica Sant Pau (IIB Sant Pau), Barcelona, Spain; 5 Grup de Neurofarmacologia Molecular, Institut de Neurociències, Universitat Autònoma de Barcelona, Barcelona, Spain; 6 Grup de Bioquímica Cardiovascular, Institut de Recerca de l’Hospital de la Santa Creu i Sant Pau & Institut d’Investigació Biomèdica Sant Pau (IIB Sant Pau), Barcelona, Spain; 7 Departament de Bioquímica, Hospital de la Santa Creu i Sant Pau, Barcelona, Spain; 8 Departament de Patologia, Hospital de la Santa Creu i Sant Pau, Barcelona, Spain; 9 Departament de Ciències Morfològiques, Universitat Autònoma de Barcelona, Barcelona, Spain; Centro Cardiologico Monzino, ITALY

## Abstract

The antinociceptive effects of the carbon monoxide-releasing molecule tricarbonyldichlororuthenium (II) dimer (CORM-2) during chronic pain are well documented, but most of its possible side-effects remain poorly understood. In this work, we examine the impact of CORM-2 treatment on the lipoprotein profile and two main atheroprotective functions attributed to high-density lipoprotein (HDL) in a mouse model of type 1 diabetes while analyzing the effect of this drug on diabetic neuropathy. Streptozotocin (Stz)-induced diabetic mice treated with CORM-2 (Stz-CORM-2) or vehicle (Stz-vehicle) were used to evaluate the effect of this drug on the modulation of painful diabetic neuropathy using nociceptive behavioral tests. Plasma and tissue samples were used for chemical and functional analyses, as appropriate. Two main antiatherogenic properties of HDL, i.e., the ability of HDL to protect low-density lipoprotein (LDL) from oxidation and to promote reverse cholesterol transport from macrophages to the liver and feces in vivo (m-RCT), were also assessed. Stz-induced diabetic mice displayed hyperglycemia, dyslipidemia and pain hypersensitivity. The administration of 10 mg/kg CORM-2 during five consecutive days inhibited allodynia and hyperalgesia and significantly ameliorated spinal cord markers (*Cybb* and *Bdkrb1*expression) of neuropathic pain in Stz mice, but it did not reduce the combined dyslipidemia shown in Stz-treated mice. Its administration to Stz-treated mice led to a significant increase in the plasma levels of cholesterol (∼ 1.4-fold *vs*. Ctrl, ∼ 1.3- fold *vs*. Stz-vehicle; *p <* 0.05) and was attributed to significant elevations in both non-HDL (∼ 1.8-fold *vs*. Ctrl; ∼ 1.6-fold *vs*. Stz-vehicle; *p <* 0.05) and HDL cholesterol (∼ 1.3-fold *vs*. Ctrl, ∼ 1.2-fold *vs*. Stz-vehicle; *p <* 0.05). The increased HDL in plasma was not accompanied by a commensurate elevation in m-RCT in Stz-CORM-2 compared to Stz-vehicle mice; instead, it was worsened as revealed by decreased [^3^H]-tracer trafficking into the feces in vivo. Furthermore, the HDL-mediated protection against LDL oxidation ex vivo shown by the HDL isolated from Stz-CORM-2 mice did not differ from that obtained in Stz-vehicle mice. In conclusion, the antinociceptive effects produced by a high dose of CORM-2 were accompanied by antioxidative effects but were without favorable effects on the dyslipidemia manifested in diabetic mice.

## Introduction

Painful diabetic neuropathy is a common complication in humans, with approximately one-third of diabetic patients displaying this important clinical problem [1–4], and new alternatives for alleviating painful neuropathy are necessary.

It is well known that dying back-type distal axon degeneration is a common underlying characteristic associated with diabetic neuropathy [5]. Nerve dysfunction and degeneration are thought to lead to sensory deficits, reduced nerve conduction velocities, and decreased epidermal innervation, all of which are characteristic signs of diabetic neuropathy in both patients and animal models [6,7]. On the other hand, the contribution of spinal cord inflammation as an emerging concept in the progression of painful diabetic neuropathy has also been reported [8].

Diabetic rodent models, including those induced by streptozotocin (Stz), exhibit the key features of human pathology, including painful neuropathy [9,10], dyslipidemia [11] and a significant upregulation of *Cybb* and *Bdkrb1* in the spinal cord, which are two specific key molecular targets directly associated with increased hyperglycemia-induced oxidative stress [12,13] and inflammation [8,14–16]. Thus, *in vivo* evaluation of the underlying mechanisms leading to oxidative stress in the development of neuropathy and the screening of novel potential therapies are plausible [10,17].

Current pharmacological treatments have proven ineffective in the alleviation of neuropathic pain [2]. The use of carbon monoxide (CO) holds great promise as a therapeutic agent [18]. Its use clearly requires a pharmaceutical formulation that allows for the delivery and/or local release of CO from a nontoxic pro-drug. In this regard, carbon monoxide-releasing molecules (CO-RMs) have been put forward as a valid alternative to the CO gas-based therapies shown to exert substantial protective effects similar to those produced by CO inhalation in different animal models of disease [18]. Particularly, tricarbonyldichlororuthenium (II) dimer (CORM-2), one of the widely used representatives of CO-RMs, has been therapeutically and satisfactorily tested in experimental models of neuropathic pain. Indeed, the repetitive administration of CORM-2 attenuated nociceptive symptoms in several nerve injury-induced types of neuropathic pain, for which the effects are mainly mediated by reducing the expression of CD11b/c (a marker of activated microglia) as well as of several oxidonitrosative stress markers [19–21]. Moreover, it was recently reported that acute administration of CORM-2 also attenuated nociceptive symptoms in diabetic mice [10].

Diabetes is a chronic pro-inflammatory condition [22], and the molecular mechanisms involved in the antioxidant and anti-inflammatory effects of CORM-2 on diabetic neuropathy have not yet been explored. Thus, we tested the hypothesis that the antinociceptive effects of CORM-2 would be directly accompanied by a concomitant attenuation of the gene expression of specific markers of oxidative stress and inflammation in the spinal cord of diabetic mice.

Diabetes mellitus is frequently accompanied by dyslipidemia due to disturbances in plasma lipids, which in turn may predispose patients to precocious cardiovascular disease [23]. Strong evidence indicates that the main atheroprotective properties of high-density lipoprotein (HDL), i.e., macrophage-to-feces reverse cholesterol transport (m-RCT) in vivo and HDL antioxidative activity are disabled in diabetes mellitus [24–26]. HDL is a class of plasma lipoproteins and has many biological roles [27]. In particular, the promotion of m-RCT is considered a major mechanism responsible for the HDL-mediated atheroprotection in vivo. This complex pathway comprises several critical steps. The first step is cholesterol efflux from lipid-laden macrophages to HDL, followed by transfer of the cholesterol to the liver, from where it may be partly eliminated via bile into the intestine and ultimately to the feces [28]. Cholesterol efflux from macrophages to plasma acceptors is the first step of this pathway and is mainly mediated via two cholesterol transporter ATP-binding cassettes, (Abc)a1 and Abcg1. After HDL remodeling in the plasma, the liver takes up HDL-derived cholesterol via the SR-BI receptor. The passage of hepatic cholesterol into bile is mainly regulated by the cholesterol canalicular transporters Abcg5/g8 or by the apical bile salt export pump (Abcb11) after being converted into bile acid by the microsomal cytochrome p450 enzyme cholesterol 7α-hydroxylase (Cyp7a1). The intestinal ABC transporters (Abcg5/g8) also take part in the regulation of the fecal excretion of cholesterol, whereas Niemann–Pick C1-like 1 (Npc1l1) is essential for the intestinal reabsorption of cholesterol [28].

The ability to attenuate the process of low-density lipoprotein (LDL) oxidation is another major antiatherogenic function of HDL [29]. This action is partly achieved by HDL-associated enzymes, including paraoxonase (PON)1. These enzymes contribute to detoxifying LDL from oxidized phospholipids and are dysfunctional during metabolic diseases, including diabetes mellitus [30]. Serum concentrations of PON1 are decreased in subjects with diabetes mellitus type 1 and feature the ability of HDL to protect LDL from oxidation [24]. Thus, we also tested the hypothesis that two of the most important anti-atherogenic properties attributed to HDL, i.e., in vivo m-RCT and antioxidant protection, which are compromised in diabetic mice, would be modified by the administration of CORM-2.

## Materials and methods

### Animals

In vivo experiments were performed in male C57BL/6NCrl mice obtained from Charles River Laboratories International, Inc. (strain code 027; Bar Harbor, ME). All mice were between 8 to 10 weeks old, weighed 21 to 25 g and were housed under 12-h/12-h light/ dark conditions in a room with controlled temperature (22°C) and humidity (66%). Animals had free access to food and water and were used after a minimum of 6 days of acclimatization to the housing conditions. All experiments were conducted between 9:00 AM and 5:00 PM. Mice were exsanguinated directly from the heart at the end of the procedure, and blood was placed into tubes containing anticoagulant (1 mM ethylenediamine-tetraacetic acid, EDTA) and serum tubes. Plasma and serum were stored at -70°C before analysis. All tissues, including lumbar sections of the spinal cord, were removed after euthanizing the animals by cervical dislocation, frozen in liquid nitrogen and stored at -70°C. Food and water intake was monitored in age-matched, individually housed mice using wire-bottomed cages for a period of 48 h. Food intake measurement was performed before (by preweighing a known amount of food) and after (by weighing the remaining food) this period of time. All experimental procedures within this study were carried out in accordance with the Directives of the European Union Council (2010/63/UE) and of the Spanish Government (RD 53/2013) for the use of animals in research. Furthermore, the protocol was approved by the local Ethical Committee of our institution (Comissió d’Ètica en l’Experimentació Animal i Humana de la Universitat Autònoma de Barcelona). All efforts were made to minimize animal suffering and to reduce the number of animals used.

### Induction of painful diabetic neuropathy and treatment

Diabetes was induced by the intraperitoneal administration of five consecutive daily injections of 55 mg/kg streptozotocin (Stz; Sigma-Aldrich, St. Louis, MO) freshly prepared in citrate buffer (0.1 M, pH 4.5). Diabetes was assessed by measuring blood glucose levels using an AccuCheck glucometer (Roche diagnostics). After injection, Stz animals were randomly distributed into three groups (n = 6 in each group) and given an intraperitoneal administration of 5 or 10 mg/kg CORM-2 solution (Stz-CORM-2) dissolved in 1% dimethylsulfoxide (DMSO; Sigma-Aldrich, St. Louis, MO) in saline or vehicle (Stz-vehicle) (1% DMSO in saline) during five consecutive days from day 21 to 25 after Stz injection. A non-diabetic group treated with citrate (Ctrl-vehicle) was used as a control. The number of 6 animals per group was determined from a pilot study taking into account a value of α = 0.01 and β = 0.10. The doses and time points of CORM-2 chosen were selected from preliminary experiments shown to be effective in producing neuropathic pain relief [9,31] as well as from our previous pilot studies performed in the Stz-induced diabetic neuropathy mouse model.

### Nociceptive behavioral tests

The development of mechanical allodynia, thermal hyperalgesia, and thermal allodynia was assessed using von Frey filaments, plantar test, and cold plate test, respectively, as described [32].

Mechanical allodynia was quantified by measuring the hind paw withdrawal response to von Frey filament stimulation. In brief, animals were placed in methacrylate cylinders (20 cm high, 9 cm diameter; Servei Estació, Barcelona, Spain) with a wire grid bottom through which the von Frey filaments (North Coast Medical, Inc., San Jose, CA) with a bending force in the range of 0.008–3.5 g were applied using a modified version of the up-down paradigm, as previously reported [33]. Clear paw withdrawal, shaking, or licking of the paw was considered a nociceptive-like response. Animals were habituated for 1 h before testing to allow appropriate behavioral immobility.

Thermal hyperalgesia was assessed as previously reported [34]. Paw withdrawal latency in response to radiant heat was measured using the plantar test apparatus (Ugo Basile, Varese, Italy). Briefly, mice were placed in methacrylate cylinders (20 cm high x 9 cm diameter) positioned on a glass surface. The heat source was positioned under the plantar surface of the hind paw and activated with a light beam intensity. The mean paw withdrawal latencies were determined from the average of three separate trials taken at 5 min intervals to prevent thermal sensitization and behavioral disturbances. Animals were habituated to the environment for 1 h before the experiment to encourage quiet and to allow testing.

Thermal allodynia to cold stimulus was assessed using the cold plate analgesia meter (Ugo Basile) as previously described [35]. The number of elevations of the hind paw was recorded in mice exposed to the cold plate (4.0 ± 0.5°C) for 5 min.

Behavioral tests were performed 3 hours after drug administration by an experimenter blinded to the treatment applied.

### Laboratory methods

#### Biochemical analyses

The methods used for plasma analyses have been described in detail elsewhere [36]. Most plasma chemicals such as, cholesterol (cat#3039773190); triglycerides (cat#20767107322); glucose (cat#4404483190); creatinine (cat#04810716190), alanine aminotransferase (ALT, cat#20764949322) and aspartate aminotransferase (AST, cat#20764949322) activities were determined using commercial kits adapted to a COBAS c501 autoanalyzer (Roche Diagnostics, Madrid, Spain). Free cholesterol (cat#435–35801), phospholipids (cat#296–63801), and free fatty acids (FFA, NEFA-HR1: cat#434–91795; NEFA-HR2: cat#436–91995) were determined using reagents from Wako Diagnostics. Triglyceride determinations were corrected for the free glycerol present in plasma (cat#F6428-40ML; Sigma-Aldrich St. Louis, MO). Quality control testing for each of these assays was done at run-time and all of them fell within the acceptable range defined as 2 times standard deviation. Precicontrol clin chem Multi 1 (cat# 5117208922, Roche diagnostics) and Precicontrol clin chem Multi 2 (cat# 5117291922, Roche diagnostics) and (cat#410–00102, Wako Chemicals). The calculated coefficient of variation for each assay was ≤ 10%. Glycated hemoglobin (HbA1c) was determined in EDTA-blood samples by automated cation exchange HPLC using BIORAD VARIANT II turbo (cat# 270-2455EX; Bio-Rad Laboratories, S.A., Madrid, Spain,). HDL cholesterol was measured in apolipoprotein (Apo)B-depleted plasma, obtained after precipitation with phosphotungstic acid and magnesium ions (Roche Diagnostics).

#### Preparation of lipoproteins

Lipoprotein fractions (HDL) in all groups of mice and human LDL were isolated from pooled plasma by sequential ultracentrifugation at 100,000 g for 24 h in an analytical fixed-angle rotor (50.3, Beckman Coulter). HDL was isolated at a density range between 1.063 and 1.21 g/mL, whereas human LDL obtained from normolipidemic volunteers was isolated from plasma-EDTA at a density range between 1.019 and 1.063 g/mL. The obtention and use of human plasma samples for this study were approved by the Ethics Committee of the Hospital de la Santa Creu i Sant Pau in Barcelona. The composition of the lipoprotein subfraction was analyzed for lipids, including total cholesterol, free cholesterol, phospholipids, and triglycerides, and proteins (total protein content) at the indicated times using commercial methods as described above. The protein levels in isolated lipoprotein fractions were determined using the bicinchoninic acid assay (cat#23225; Thermo Fisher Scientific, Waltham, Massachusetts).

#### Histology

Formalin-fixed epididymal adipose tissues were embedded in paraffin, and samples were sectioned at 5 μm for hematoxylin and eosin (H&E) staining [37].

#### Fecal and liver lipid analyses

Stools from individually housed mice, fed ad libitum and with free access to water, were collected over 2 days. Mice were euthanized and exsanguinated by cardiac puncture at the end of the study and livers removed after extensive perfusion with saline. Lipids were extracted with isopropyl alcohol-hexane (2:3; v:v) from 1 g of feces and 100 mg of liver. After the addition of sodium sulfate (Na_2_SO_4_), the hexane phase was isolated, dried with nitrogen, reconstituted with 0.5% sodium cholate, and sonicated using an ultrasound bath (model 5510-MT, Branson Ultrasonics Corp., Danbury, CT, USA) for 10 min (50 Hz) prior to lipid measurements.

#### Measurement of m-RCT in vivo

[^3^H]-cholesterol-labeled J774 macrophages were prepared and intraperitoneally injected into mice, as described previously [38]. Mice were then individually housed in metabolic cages, and stool was collected over the next two days. Plasma radioactivity was determined at 48 h by liquid scintillation counting. HDL-associated [^3^H]-cholesterol was measured after the precipitation of ApoB-containing lipoproteins with phosphotungstic reagent. At this point, mice were euthanized and livers collected. Liver and fecal lipids were extracted with isopropyl alcohol-hexane. The lipid layer was collected, evaporated, and [^3^H]-cholesterol radioactivity measured by liquid scintillation counting. The [^3^H]-tracer detected in fecal biliary acids was determined in the remaining aqueous phase of the fecal material extracts. The amount of [^3^H]-tracer was expressed either as a fraction of the injected dose or as relative to Ctrl-vehicle, which were taken as 100%.

#### Susceptibility to lipoprotein oxidation

Isolated lipoproteins were dialyzed in phosphate-saline buffer by gel filtration on PD-10 columns (Sephadex G-25™ M, cat#17-0851-01; GE Healthcare). Oxidation was initiated by adding 2.5 μmol/L copper (II) sulfate (CuSO_4_) to human LDL (0.1 mmol/L phospholipids) and mouse HDL (0.1 mmol/L of phospholipids) mixtures or to human LDL and mouse HDL alone [39]. Conjugated diene formation was measured by continuous monitoring of absorbance at 234 nm in a Synergy HT microplate reader (BioTek Synergy) at 37°C for 6 h. The lag phase was calculated from the intersection point between the maximal slope of the curve and initial absorbance, as previously described [39]. Three oxidation kinetics were assayed: human LDL alone, mouse HDL alone, and the mixture of human LDL plus mouse HDL (hLDL+mHDL). The kinetics of human LDL in the presence of mouse HDL was calculated by subtracting the kinetics of mouse HDL alone from the mixture hLDL+mHDL. The antioxidant capacity of each HDL was the ability to prolong the lag phase and was expressed as the increment versus Ctrl-vehicle mice. The longer the lag phase the higher the antioxidant capacity of HDL.

Total serum arylesterase activity was measured using phenyl acetate as a substrate [39]. EDTA-sensitive serum arylesterase activity was calculated by subtracting the EDTA-resistant arylesterase and expressed as paraoxonase (PON)-1 activity. The latter was determined using 1 mmol/L EDTA in the reaction buffer instead of calcium chloride (CaCl_2_), which was used to determine total arylesterase activity [40].

#### Quantitative real-time RT-PCR analyses

Spinal cord, liver, and small intestine ribonucleic acid (RNA) was isolated using the TRIzol RNA isolation method (Ambion, cat# 15596018; Life technologies, Carlsbad, CA, USA). Total RNA samples were then repurified (RNeasy mini kit Plus, cat# 74134; Qiagen, CA, USA). A ratio of the absorbances at 260 and 280 nm was used to assess the purity of RNA. A ratio of ∼2.0 is generally accepted as “pure” for RNA. The purities of the total RNA samples were determined using a Nano-drop UV/Vis, ND-1000 spectrophotometer and NanoDrop software (version 3.5.2) (Nano-drop Technologies, Inc., Wilmington, DE, USA). Total mRNA (1 μg) was reverse-transcribed with Oligo(dT)_15_ using M-MLV Reverse Transcriptase, RNase H Minus, and Point Mutant (Promega Corporation, MD, USA) to generate cDNA. Predesigned validated primers (Assays-on-Demand; Applied Biosystems, Foster City, CA) were used with TaqMan probes. Specific mouse Taqman probes (Applied Biosystems) were used for *Abca1* (Mm00442646_m1), *Abcb11* (Mm00445168_m1), *Abcg1* (Mm00437390_m1), *Abcg5* (Mm00446241_m1), *Abcg8* (Mm00445970_m1), *Apoa1* (Mm00437569_m1), *Bdkrb1* (Mm04207315_s1), *Cd36* (Mm01135198_m1), *Cybb* (Mm01287743_m1), *Cy7a1* (Mm00484152_m1), *Lipe* (Mm00495359_m1), *Lpl* (Mm00434764_m1), *Ncp1l1* (Mm01191972_m1), *Pon1* (Mm00447161_m1), *Scarb1* (Mm00450236_m1), and *Gapdh* (Mm99999915_g1). Real-time PCR assays were performed on a C1000 Thermal Cycler coupled to a CFX96 Real-Time System (Bio-Rad Laboratories SA, Life Science Group, Madrid, Spain). All analyses were performed in duplicate. *Gapdh* was used as the reference gene in the liver, small intestine and adipose tissues, whereas 18S was used to normalize the mRNA levels in the spinal cord. The relative mRNA expression levels were calculated by the ΔΔCt method.

### Statistical analysis

The data are expressed as the mean ± standard error of the mean (SEM). For behavioral studies, the data were analyzed using repeated measures two-way ANOVA followed by the corresponding one-way ANOVA and Student-Newman-Keuls test.

The effects of diabetes or CORM-2 treatment on gross and plasma chemical parameters, kinetic studies, and HDL properties or the levels of gene expression were determined using either a non-parametric Kruskal-Wallis test followed by a Dunn test or parametric one-way ANOVA followed by a Student-Newman-Keuls test, as appropriate.

The relationship between variables was tested with Pearson’s correlation. Statistical analyses were performed using SPSS (version 17 for Windows; IBM España, Madrid, Spain) and GraphPad Prism software (GPAD, version 5.0, San Diego, CA, USA). A value of *p <* 0.05 was considered significant.

## Results

### Treatment with CORM-2 prevents the development of painful diabetic neuropathy in mice

**Fig 1:**
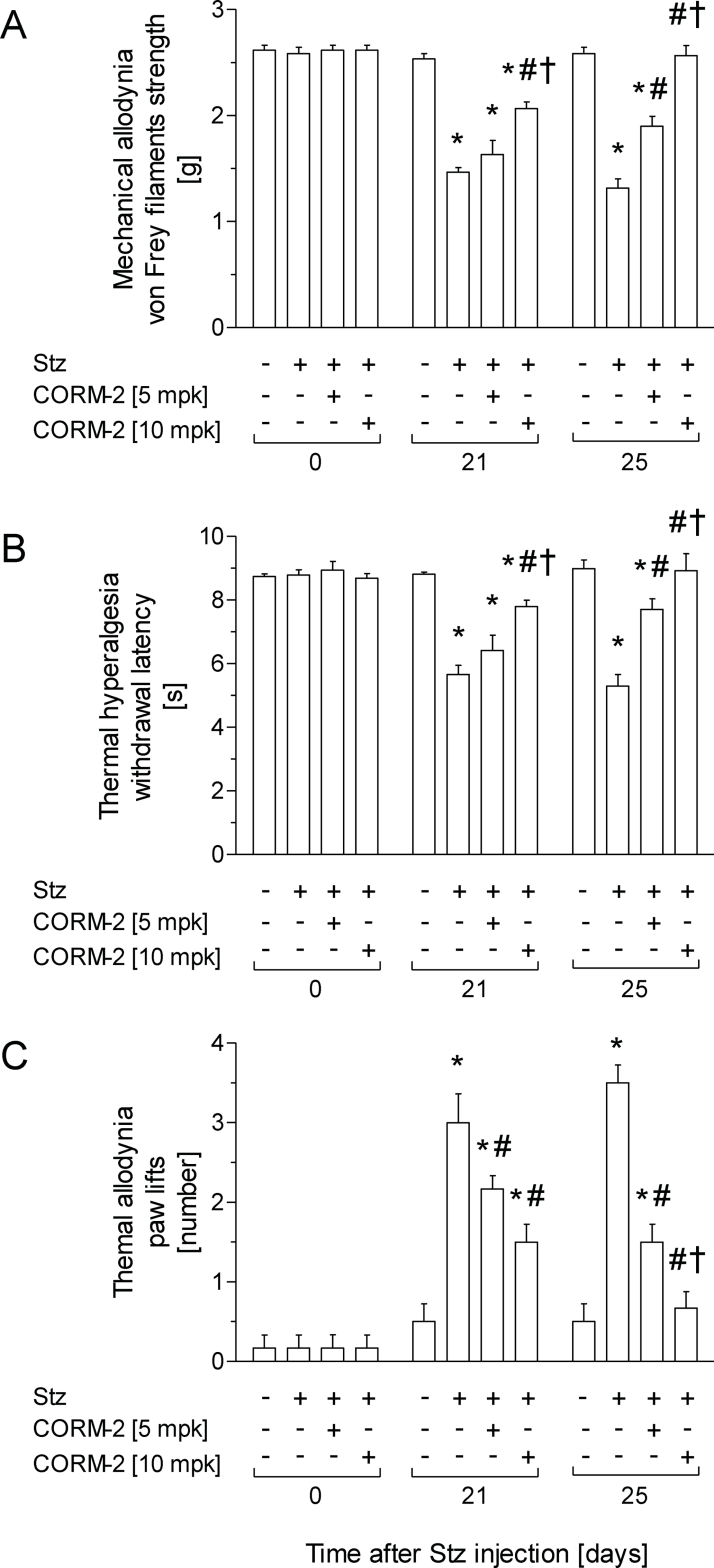
The administration of CORM-2 alleviates signs of neuropathic pain displayed by diabetic mice. Antinociceptive effects produced by the intraperitoneal administration of CORM-2 at 5 or 10 mg/kg in STZ injected mice. Results are shown as the mean values ± SEM; n = 6 animals per group. The development of mechanical allodynia (A), thermal hyperalgesia (B) and thermal allodynia (C) in the hind paws of control and diabetic mice intraperitoneally treated with vehicle or CORM-2 at 5 or 10 mg/kg is represented. Data of tests are shown at day 0 (before diabetes induction) and at days 21 and 25 after Stz injection (one and five days after initiation of CORM-2 administration, respectively). For each test and day, * indicates significant differences *vs*. Ctrl-vehicle, # *vs*. Stz-vehicle treated mice and † *vs*. Stz mice treated with 5 mg/kg CORM-2 (*p <* 0.05, one-way ANOVA followed by a Student-Newman-Keuls test). CORM-2, (tricarbonyldichlororuthenium(II) dimer; Ctrl, control mice; Stz, streptozotocin treated mice.

For mechanical allodynia (Fig 1A), thermal hyperalgesia (Fig 1B) and thermal allodynia (Fig 1C) induced by Stz, two-way ANOVA repeated measures revealed a significant effect of treatment (*p <* 0.001), time (*p <* 0.001), and its interaction (*p <* 0.001). Indeed, while mechanical allodynia and thermal hyperalgesia induced by Stz were reduced in animals treated with CORM-2 at 10 mg/kg for one day (*p <* 0.001; one-way ANOVA vs. Stz-vehicle animals), both nociceptive responses were significantly reduced in animals treated with 5 mg/kg CORM-2 for five days (*p <* 0.001; one-way ANOVA vs. Stz-vehicle) or completely inhibited by the administration of 10 mg/kg CORM-2 for five days (*p <* 0.001; one-way ANOVA vs. Stz-vehicle). Moreover, the mechanical antiallodynic and thermal antihyperalgesic effects produced by CORM-2 administered at 10 mg/kg were significantly higher (*p <* 0.001; one-way ANOVA) than those produced by the administration of 5 mg/kg CORM-2 for one or five consecutive days.

For the thermal allodynia induced by Stz, while it was similarly inhibited in animals treated with CORM-2 at 5 or 10 mg/kg for one day (*p <* 0.001; one-way ANOVA vs. Stz-vehicle animals), the thermal antiallodynic effects produced by 10 mg/kg CORM-2 for five days were significantly higher (*p <* 0.001; one-way ANOVA) than those produced by this compound at 5 mg/kg for five days of treatment. Indeed, while the thermal allodynia induced by Stz was completely inhibited by treatment with CORM-2 at 10 mg/kg for 5 days, it was only significantly alleviated in CORM-2-treated mice at 5 mg/kg for 5 days (*p <* 0.001; one-way ANOVA vs. Ctrl animals).

In Ctrl mice, CORM-2 treatment did not produce any effects compared to mice treated with vehicle (data not shown).

**Fig 2:**
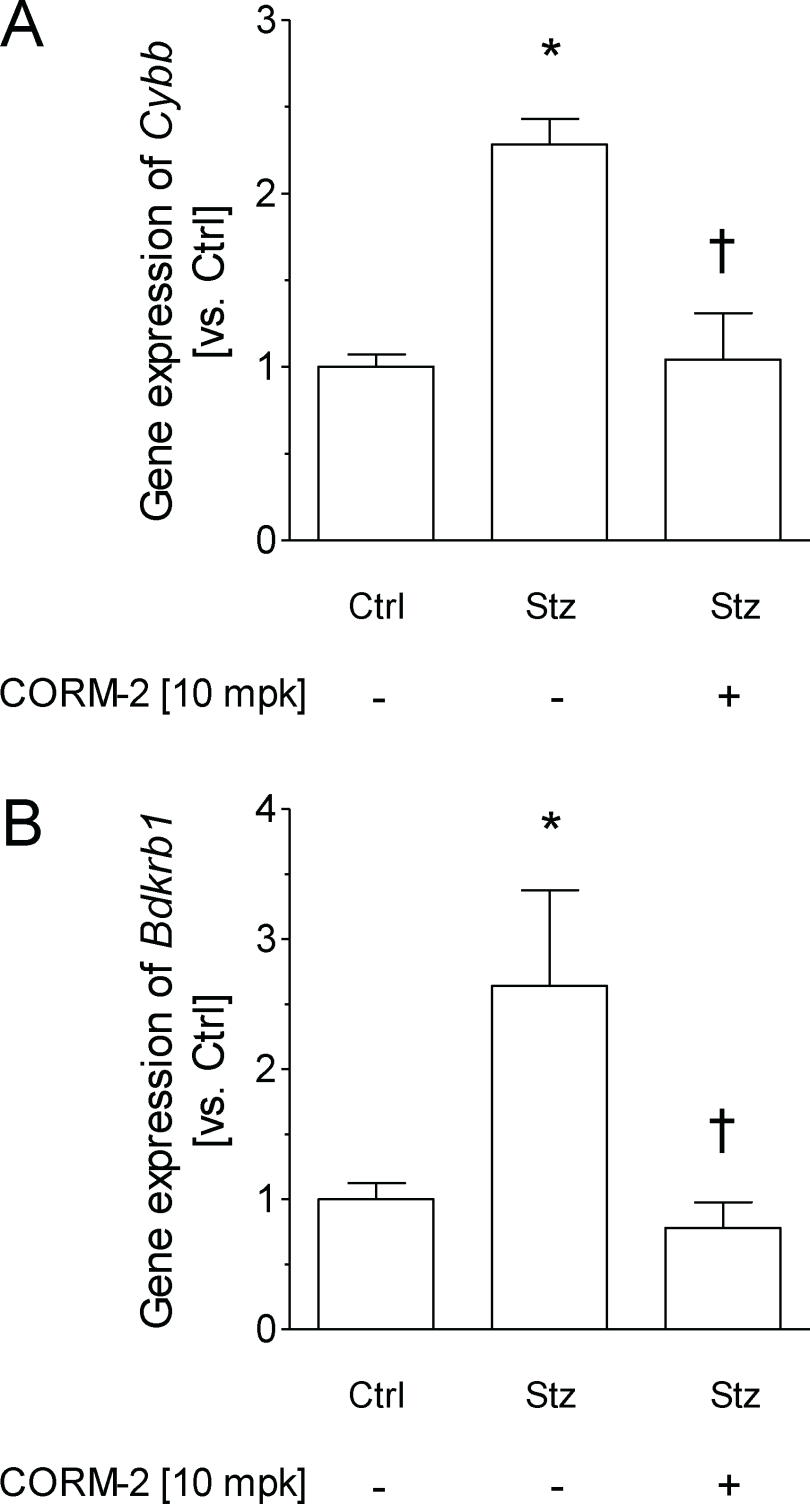
CORM-2 treatment down-regulates the gene expression of Nox2 and B1R in the spinal cord from STZ treated animals. The relative gene expression of *Cybb* (Nox2) (A) and *Bdkrb1* (kinin B1 receptor) (B) in the spinal cord of Ctrl and diabetic mice treated with vehicle or 10 mg/kg CORM-2 for five consecutive days are represented. Data are expressed as the mean ± SEM of n = 5 samples per group. For all panels, * indicates significant differences *vs*. Ctrl-vehicle and † *vs*. Stz-vehicle treated mice (*p <* 0.05, one-way ANOVA followed by a Student-Newman-Keuls test). CORM-2, (tricarbonyldichlororuthenium(II) dimer; Ctrl, control mice; Stz, streptozotocin treated mice.

Because spinal cord inflammation has been suggested to underlie the pathophysiology of neuropathic pain [8], we assessed the gene expression of *Cybb* (i.e., Nox2), a recognized marker of oxidative stress, and *Bdkrb1* (i.e., bradykinin B1 receptor, B1R), a marker of inflammation, in the spinal cords of Stz-vehicle mice (Fig 2). Gene expression of both gene targets was significantly upregulated in Stz-vehicle mice (*Cybb*: ∼ 2.2-fold vs. Ctrl-vehicle, *p <* 0.003; *Bdkrb1*: ∼ 2.6-fold vs. Ctrl-vehicle; *p <* 0.021) (Fig 2A and 2B). Notably, the diabetes-induced upregulation of both gene targets was completely inhibited by CORM-2 treatment. Moreover, as shown in S1 Table, the relative gene expression of *Cybb* and *Bdkrb1* was directly correlated with the mechanical allodynia, thermal hyperalgesia and thermal allodynia induced by STZ.

### Administration of CORM-2 does not improve carbohydrate metabolism in diabetic mice

Body weight and fat content were significantly lower in Stz mice than in control mice (*p <* 0.05, Table 1). As expected, both the food and water intake were increased in diabetic mice. Although the absolute liver weight did not differ among groups, the relative liver weight (expressed vs. total body weight) was increased in both groups of Stz-treated mice (Table 1). The plasma levels of ALT in Stz-vehicle mice were elevated (∼ 2-fold, *p <* 0.05) compared with Ctrl-vehicle mice (Table 1), while no changes were observed in plasma AST levels. Administration of CORM-2 to Stz mice did not have any effect on either the body weight or main liver lipids. However, the Stz-CORM-2 mice had lower plasma levels of ALT than the Stz-vehicle mice.

Unexpectedly, the increase in the daily caloric intake of Stz-CORM-2 mice was even more pronounced than that of Stz-vehicle mice (Table 1). This finding was concomitant with a significant reduction in epididymal adipose tissue in these mice compared to Stz-vehicle mice. Adipocyte size was reduced in diabetic mice compared with Ctrl-vehicle mice (S1 Fig). No changes in the gene expression of hormone-sensitive lipase (*Lipe*) or lipoprotein lipase (*Lpl*), both of which determine triglyceride stores in adipose tissue [41], were revealed in epididymal adipose tissue between the two groups of diabetic mice (S2 Table).

**Table 1:** Gross, metabolic data, biochemical parameters in plasma, and hepatic lipids in mice on day 25 after injection of Stz or citrate.

	*Ctrl*	*Stz*		
*Parameters*	*Vehicle*	*vehicle*	*CORM-2*	*p*
**Gross**				
Final weight [g]	26.68 ± 0.61	21.62 ± 0.74 *	20.56 ± 0.77 *	< 0.05
Liver weight [g]	1.53 ± 0.06	1.53 ± 0.08	1.42 ± 0.06	0.06
Liver-to-body weight ratio	0.059 ± 0.001	0.070 ± 0.001 *	0.069 ± 0.001 *	< 0.05
Epididymal fat [g]	0.38 ± 0.05	0.11 ± 0.03 *	0.07 ± 0.01 * †	< 0.05
**Metabolic data**				
Food intake [kcal/day]	9.95 ± 0.60	20.51 ± 0.78 *	24.66 ± 1.60 * †	< 0.05
Water intake [mL/day]	6.24 ± 1.03	44.25 ± 1.44 *	44.98 ± 2.38 *	< 0.05
**Biochemical parameters in plasma**				
Glucose [mM]	11.78 ± 0.46	36.30 ± 2.53 *	36.52 ± 2.41 *	< 0.05
Insulin [μU/mL]	0.52 ± 0.11	0.20 ± 0.02 *	0.24 ± 0.05 *	< 0.05
HbA1c [%]	3.4 ± 0.0	5.2 ± 0.3 *	4.6 ± 0.5 *	< 0.05
ALT [U/L]	28.04 ± 6.19	60.00 ± 7.85 *	49.77 ± 3.68	< 0.05
AST [U/L]	104.30 ± 24.02	291.70 ± 40.43	210.7 ± 37.77	0.69
Creatinine [μM]	24.7 ± 1.1	41.1 ± 1.5 *	44.7 ± 4.3 *	< 0.05
Total cholesterol [mM]	3.18 ± 0.03	3.39 ± 0.10	4.49 ± 0.11 * †	< 0.05
HDL cholesterol [mM]	2.41 ± 0.07	2.46 ± 0.08	3.08 ± 0.09 * †	< 0.05
Non-HDL cholesterol [mM]	0.77 ± 0.04	0.91 ± 0.07	1.42 ± 0.16 *	< 0.05
Total triglycerides [mM]	0.88 ± 0.22	3.03 ± 0.85 *	4.61 ± 1.57 *	< 0.05
Total FFA [mM]	0.67 ± 0.03	1.07 ± 0.11 *	1.36 ± 0.16 *	< 0.05
**Hepatic lipids**				
Liver cholesterol [μmol/g]	1.52 ± 0.12	1.61 ± 0.11	1.72 ± 0.11	0.73
Liver triglycerides [μmol/g]	2.19 ± 0.54	2.60 ± 0.46	2.79 ± 0.79	0.82

Data are expressed as the mean ± SEM (n = 5 mice per group). Differences between the mean values were determined using either a nonparametric Kruskal-Wallis test followed by a Dunn test or parametric one-way ANOVA followed a Student-Newman-Keuls test, as appropriate.

* indicates significant differences *vs*. Ctrl-vehicle and

† *vs*. Stz-vehicle treated mice (*p* < 0.05).

ALT, alanine aminotransferase; AST, aspartate aminotransferase; CORM-2, (tricarbonyldichlororuthenium(II) dimer; Ctrl, control mice; HbA1C, glycated hemoglobin; FFA, free fatty acids; Stz, streptozotocin treated mice.

The blood glucose concentration increased significantly one week after the last injection in mice treated with Stz, and they remained diabetic throughout the 25 days of the experiment (∼ 3-fold; *p <* 0.05) (Table 1). Blood HbA1c levels were significantly increased in Stz mice on day 25 (Table 1). The severe decrease observed in insulin levels (*p <* 0.05, Table 1) revealed the Stz-induced loss of β-pancreatic cells. Administration of CORM-2 to Stz mice did not improve insulin of glucose levels (Table 1).

### CORM-2 aggravates the hyperlipemia induced by diabetes mellitus

Plasma triglycerides of Stz-vehicle mice (∼ 3-fold vs. Ctrl-vehicle; *p <* 0.05) and Stz-CORM-2 mice (Stz-vehicle: ∼ 5-fold vs. Ctrl-vehicle; *p <* 0.05) were significantly elevated compared with Ctrl-vehicle mice. Plasma FFA levels were significantly higher in Stz-vehicle (∼ 1.6-fold; *p <* 0.05) and Stz-CORM-2 (∼ 2-fold; *p <* 0.05) than in Ctrl-vehicle mice (Table 1). Plasma levels of FFA were inversely associated with a significant decrease in the epididymal fat and were directly related with plasma triglycerides and non-HDL cholesterol (S2 Fig). In this regard, the hepatic gene expression of *Cd36* was also determined and was found to be increased in Stz-treated mice (> 4-fold; *p <* 0.05) compared with Ctrl-vehicle mice (S3 Table).

Compared with Ctrl-vehicle mice, the plasma levels of total cholesterol were not significantly increased in Stz-vehicle mice (Table 1). Plasma levels of non-HDL cholesterol in these mice did not differ from those in Ctrl-vehicle mice. In contrast, the administration of CORM-2 led to a significant increase (∼ 1.3-fold *vs*. Stz-vehicle; *p <* 0.05) in the plasma levels of cholesterol due to significant elevations in both non-HDL and HDL cholesterol (Table 1). Elevated plasma levels of HDL were not accompanied by changes in their composition or particle size, the latter of which was revealed by calculated surrogate measurements (S4 Table), or altered gene expression of *Apoa1* in the livers of these mice (S5 Table).

### Administration of CORM-2 does not improve m-RCT in diabetic mice

**Fig 3:**
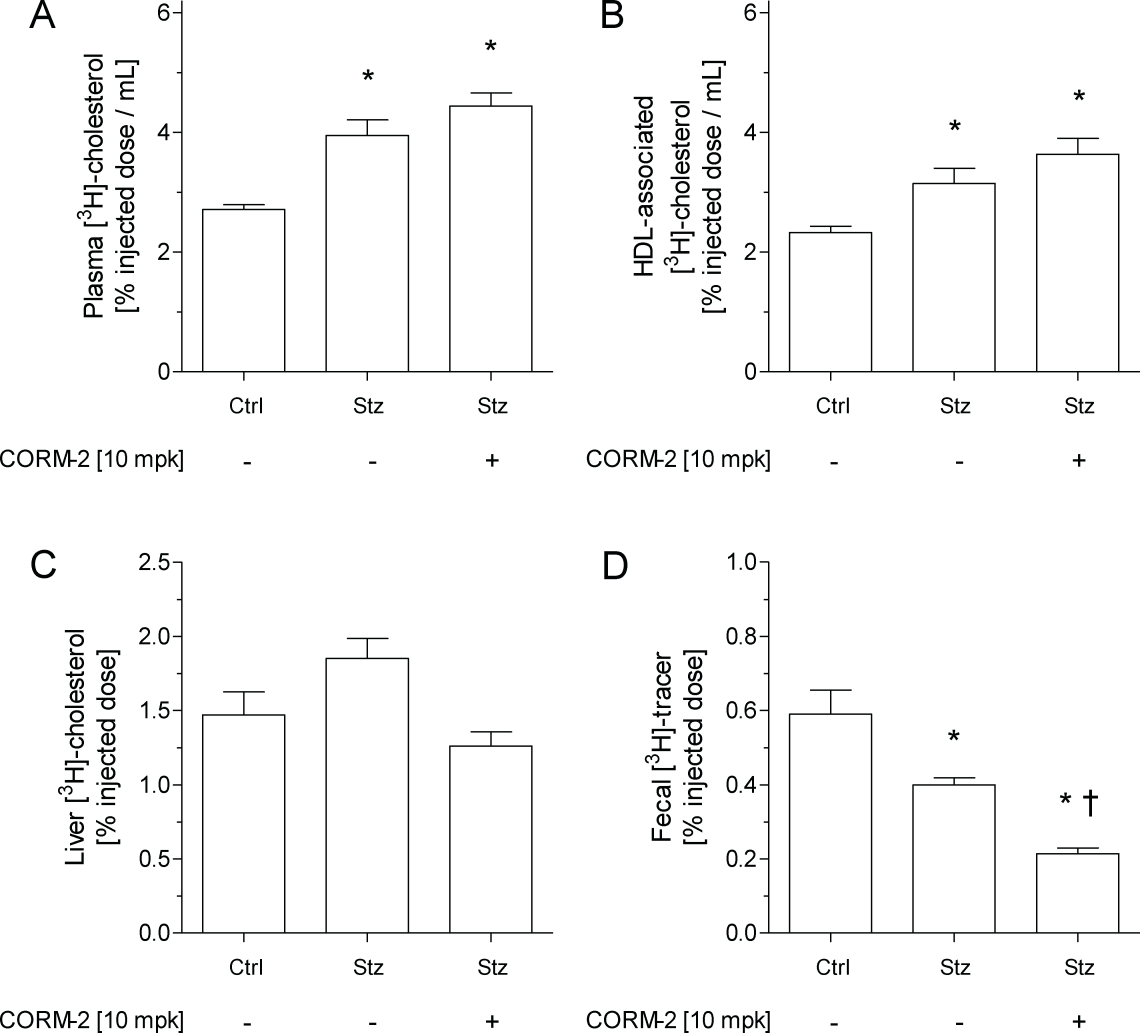
Administration of CORM-2 does not improve m-RCT in vivo in diabetic mice. Macrophage-to-plasma m-RCT was increased in diabetic mice, and liver-to-feces m-RCT was decreased in Stz-CORM-2 mice. Individually housed mice were intraperitoneally injected with 1 million [^3^H]-cholesterol-labeled J774 mouse macrophages per animal, and the distribution of counts into different compartments was determined 48 h after injection. Counts were assessed by liquid scintillation counting. At the end of the experimental period, livers were harvested, snap-frozen in liquid nitrogen, and stored at -80°C. In all panels, results are the mean ± SEM of 5 mice and are expressed in % vs. injected dose. (A) Total plasma levels of [^3^H]-cholesterol. (B) Plasma levels of [^3^H]-cholesterol in the HDL fraction. (C) Hepatic levels of [^3^H]-cholesterol. Counts in a weighed liver sample were determined following liquid extraction of the tissue and related to total liver mass. (D) Fecal [^3^H]-tracer. Feces were collected continuously up to 48 h and were dried, weighed, and thoroughly ground. Aliquots were separated into bile acid and cholesterol fractions, and counts recovered from the respective aliquots were related to the total amount of feces produced over 48 h. Differences between the mean values were determined using either a nonparametric Kruskal-Wallis test followed by a Dunn test or parametric one-way ANOVA followed a Student-Newman-Keuls test, as appropriate. For all panels, * indicates significant differences *vs*. Ctrl-vehicle and † *vs*. Stz-vehicle treated mice (*p* < 0.05). CORM-2, (tricarbonyldichlororuthenium(II) dimer; Ctrl, control mice; Stz, streptozotocin treated mice.

The analysis of m-RCT was performed to investigate whether increased HDL levels in the plasma of Stz-CORM-2 mice would translate into a favorable increase in [^3^H]-cholesterol trafficking from macrophages to the feces of diabetic mice. Stz-vehicle and Stz-CORM-2 mice exhibited higher relative levels of plasma [^3^H]-cholesterol than Ctrl-vehicle mice at 48 h after intraperitoneal injection with [^3^H]-cholesterol-loaded macrophages, with counts primarily associated with the HDL fraction (Fig 3A and 3B). The relative amount of hepatic [^3^H]-tracer present within the liver did not differ among groups (Fig 3C). However, [^3^H]-tracer counts (in the form of bile acids) in the feces of Stz-vehicle mice were lower than those of Ctrl-vehicle mice (Fig 3D and Table 2). Unexpectedly, the relative [^3^H]-tracer counts in the feces of Stz-CORM-2 mice were much lower than those found in Stz-vehicle mice (Fig 3D), which was mainly explained by a concomitant decrease observed in the fecal counts of [^3^H]-cholesterol (Table 2). The gene expression of either hepatic or small intestine targets involved in cholesterol trafficking throughout different compartments of m-RCT did not significantly differ among groups, except that the hepatic gene expression of *Abca1* was significantly downregulated in diabetic mice treated with vehicle or CORM-2 (S5 Table).

**Table 2:** Fecal [^3^H]-tracer distribution into cholesterol and bile acid fractions on an m-RCT setting.

	*Ctrl*	*Stz*		
*Parameters*	*Vehicle*	*Vehicle*	*CORM-2*	*p*
Total activity	0.59 ± 0.07	0.40 ± 0.02	0.21 ± 0.02 * †	< 0.05
Cholesterol	0.28 ± 0.03	0.30 ± 0.02	0.13 ± 0.02 * †	< 0.05
Bile acid	0.31 ± 0.05	0.10 ± 0.01 *	0.08 ± 0.01 * †	< 0.05

Data are expressed as the means (% vs. injected dose) ± SEM (n = 4–5 mice per group). Feces were collected continuously up to 48 h and were dried, weighed, and thoroughly ground. Aliquots were separated into bile acid and cholesterol fractions, and counts recovered from the respective aliquots were related to the total amount of feces produced over 48 h. Differences between the mean values were determined using either a nonparametric Kruskal-Wallis test followed by a Dunn test or parametric one-way ANOVA followed a Student-Newman-Keuls test, as appropriate.

* indicates significant differences *vs*. Ctrl-vehicle and

† *vs*. Stz-vehicle treated mice *(p <* 0.05).

CORM-2, (tricarbonyldichlororuthenium(II) dimer; Ctrl, control mice; Stz, streptozotocin treated mice.

### Administration of CORM-2 fails to improve diabetic HDL-mediated antioxidative protection against LDL oxidation ex vivo

**Fig 4:**
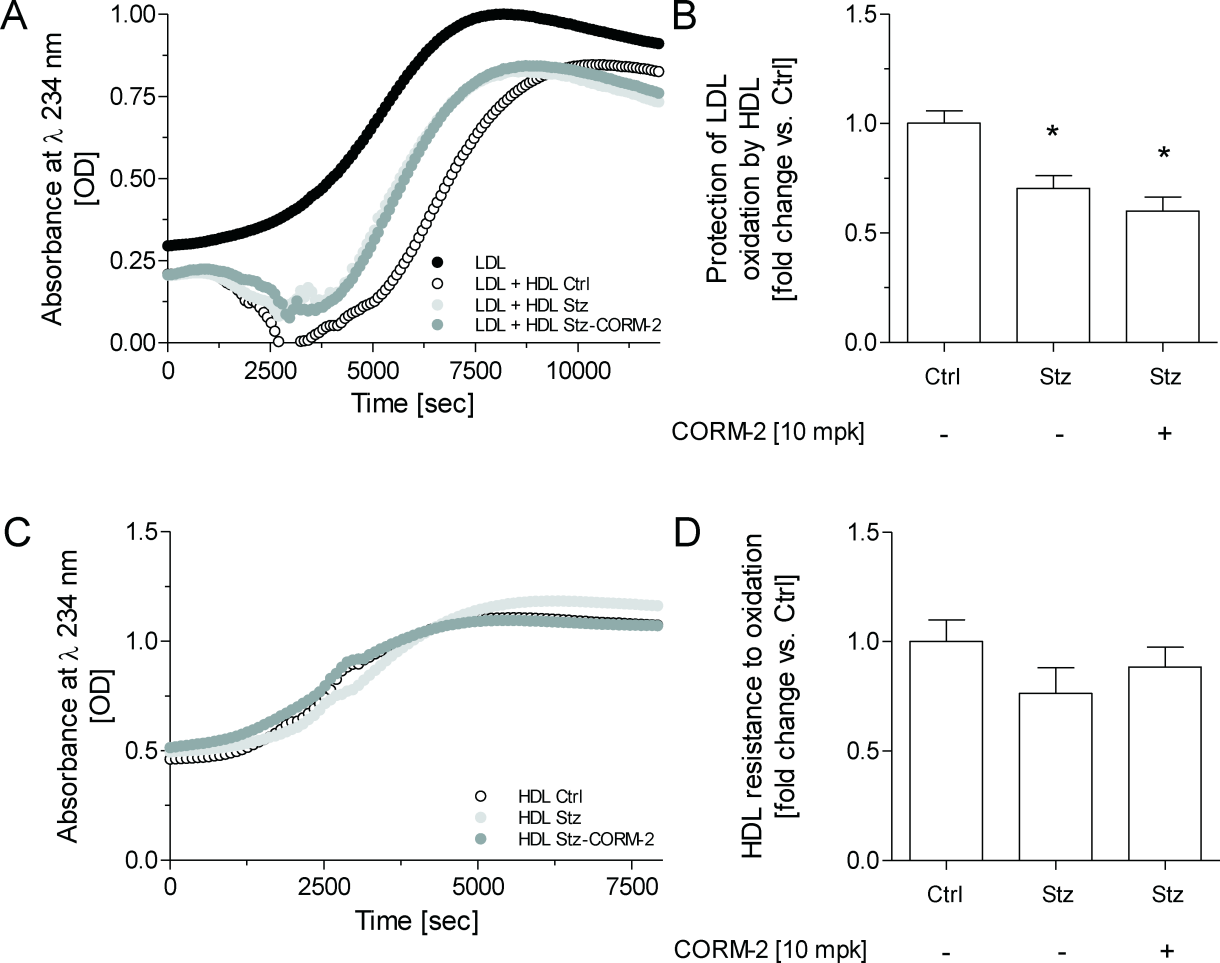
Nominal effect of CORM-2 on lipoprotein oxidation. (A) Representative diene formation curves of human LDL alone or incubated with HDL isolated from mice in the presence of 2.5 μM CuSO_4_ at 37°C. Kinetics of the LDL+HDL is shown after subtracting the kinetics of HDL incubated without LDL. (B) HDL antioxidant activity against LDL oxidative modification. Data are expressed as relative lag phase to LDL oxidized in the presence of HDL isolated from Ctrl-vehicle mice (arbitrary unit = 1). (C) Representative diene formation curves of HDL isolated from mice in the presence of 2.5 μM CuSO_4_ at 37°C. (D) HDL resistance to oxidation. Data are expressed as lag phase of conjugated diene formation kinetics represented as relative lag phase to the kinetics of oxidation of HDL from Ctrl-vehicle mice (arbitrary unit = 1). In panels B and D, the data are expressed as the mean ± SEM. Differences between the mean values were determined using either a nonparametric Kruskal-Wallis test followed by a Dunn test or parametric one-way ANOVA followed a Student-Newman-Keuls test, as appropriate. For all panels, * indicates significant differences vs. Ctrl-vehicle (*p* < 0.05). CORM-2, (tricarbonyldichlororuthenium(II) dimer; Ctrl, control mice; Stz, streptozotocin treated mice.

HDL isolated from diabetic mice showed an impaired ability to lengthen the lag phase during copper-induced LDL oxidation, compared with Ctrl-vehicle mice (Fig 4). The HDL of diabetic mice treated with CORM-2 presented a similar impaired antioxidant ability than observed in diabetic mice without CORM-2 treatment. In this regard, the plasma levels and hepatic gene expression of HDL-associated enzymes, including PON-1, were similar in Stz-vehicle and Stz-CORM-2 mice (Table 3). The plasma activity of PON-1 was slightly decreased in diabetic mice compared with non-diabetic mice (Table 3).

**Table 3:** Effect of CORM-2 on plasma levels and hepatic gene expression of PON-1.

	*Ctrl*	*Stz*		
	*Vehicle*	*vehicle*	*CORM-2*	*p*
*Plasma activity*				
Total PON-1 [μmol/mL/min]	35.8 ± 0.9	30.7 ± 2.3 *	31.5 ± 1.1 *	< 0.05
*Hepatic gene expression*				
*Pon1* [vs. Ctrl]	1.0 ± 0.1	0.8 ± 0.1	0.7 ± 0.1	0.10

Data are expressed as the mean ± SEM (n = 5 mice per group). Differences between the mean values were determined using either a nonparametric Kruskal-Wallis test followed by a Dunn test or parametric one-way ANOVA followed a Student-Newman-Keuls test, as appropriate.

* indicates significant differences *vs*. Ctrl-vehicle (*p* < 0.05).

CORM-2, (tricarbonyldichlororuthenium(II) dimer; Ctrl, control mice; PON-1, paraoxonase-1; Stz, streptozotocin treated mice.

## Discussion

The favorable antinociceptive effects of CORM-2 have been demonstrated in previous studies using different experimental models of pain, either inflammatory or neuropathic [20,32,42,43]. Consistently, our results confirmed the alleviation of diabetic neuropathy produced by the acute administration of CORM-2 [9] and further revealed that the repeated administration of a high dose of this compound completely inhibited the allodynia and hyperalgesia observed in diabetic mice.

CORM-2-derived carbon monoxide has been suggested to prevent reactive oxygen species production by directly inhibiting Nox activity [21]. Of note, we found that the administration of CORM-2 inhibited the Stz-induced overexpression of *Cybb* in the spinal cord, which may help to suppress the Nox-mediated overproduction of ROS in the spinal cords of diabetic mice. This also occurs in other pain models [44], suggesting that CORM-2 contributes to the protection against redox imbalance and oxidative injury in diabetic mice. Similarly, CORM-2 treatment also normalized the upregulation of *Bdkrb1* induced by Stz in our study. Importantly, this effect has not been previously evaluated and was associated with a significant alleviation of nociceptive symptoms. Consistently, protective actions against hyperalgesia and allodynia [14] and on the reestablishment of neuronal function [45] have been attributed to B1R antagonists in experimental models of diabetes. In agreement with our data, the specific inhibition of B1R also normalized the increased levels of several markers of oxidative stress in diabetic rats [45].

Despite the antinociceptive effects produced by CORM-2 in diabetic mice, the impact of this compound on carbohydrate and lipid homeostasis under conditions of hyperglycemia has not been previously analyzed. We describe for the first time that the administration of CORM-2 at 10 mg/kg for 5 days does not alter carbohydrate metabolism but produces an unexpected impact on fat and plasma lipids. Although it is well known that Stz leads to a significant reduction in body fat [25,46], the enhanced fat reduction promoted by CORM-2 in Stz mice has not been previously reported. Nevertheless, since energy expenditure was not directly measured in these animals, the mechanism involved in the enhanced fat reduction observed in Stz-CORM-2 mice remains unknown. Management of triglycerides is another key determinant of fat pad size, as increased plasma levels of FFA frequently reveal changes in triglyceride stores in adipose tissue [47]. Supporting this, plasma levels of FFA were inversely associated with epididymal white adipose tissue. Our data further revealed that the plasma levels of FFA or triglycerides were not significantly altered in Stz-treated mice, despite displaying a trend to be elevated. Hormone-sensitive lipase, which is activated in diabetes [41], lipoprotein lipase, which is often defective in diabetes [48], and the fatty acid transporter Cd36, which is involved in the FFA uptake [49], are regarded as main contributors of plasma levels of FFA and triglycerides. However, adipose gene expression analysis of these lipases did not show differences between both diabetic groups, it does not rule out posttranscriptional mechanisms at this level explaining the effect of CORM-2 on fat reduction. On the other hand, the hepatic mRNA levels of Cd36 were upregulated in diabetic mice but did not differ in both groups of diabetic mice. Finally, caloric intake was increased in Stz-CORM-2 compared with Stz-vehicle mice (Table 1). The mechanisms involved in this finding were not analyzed in the present work and may warrant further studies.

In relation to HDL, in contrast with a previous study [25] but consistent with another study that used the same treatment to induce diabetes [50], our data revealed that plasma levels of HDL cholesterol in Stz-vehicle mice were similar to those obtained in Ctrl-vehicle mice. We do not know the basis for this discrepancy, but it may be at least in part explained by the different treatments used to induce diabetes in each study (i.e., either Stz- or alloxan-treated mice). Importantly, the administration of CORM-2 also produced an unexpected elevation in the plasma levels of HDL. Although the metabolic basis of elevated plasma levels of HDL was not analyzed, the increased food intake observed in Stz-CORM-2 mice might potentially contribute to the raised levels of cholesterol in these mice compared with Stz-vehicle mice.

Given that elevated HDL levels may influence cardioprotection and given that their functionality is distorted in diabetes [51–54], we further examined two of the main antiatherogenic properties attributed to HDL lipoproteins, i.e., m-RCT in vivo and protection against LDL oxidation ex vivo. In relation to m-RCT, cholesterol efflux from radiolabeled macrophages and hepatic uptake of plasma radiotracer are the first steps of m-RCT [28]. [^3^H]-cholesterol efflux from macrophages to HDL from Stz-CORM-2 mice was significantly increased in a manner that was directly proportional to the concentration of HDL compared with Stz-vehicle mice (Fig 3). Hepatobiliary and fecal cholesterol trafficking are the last two components of m-RCT [28]. Consistent with previously published data [25], our diabetic mice showed a decrease in m-RCT compared with non-diabetic mice, as revealed by a significant reduction in fecal radiotracer excretion. The administration of CORM-2 to Stz mice further reduced the fecal [^3^H]-tracer excretion, which was mainly attributed to a significant decrease in the fecal [^3^H]-cholesterol component. These changes were not explained by changes in the gene expression of molecular determinants involved in the hepatobiliary cholesterol trafficking component of m-RCT. The reduced fecal [^3^H]-tracer excretion in our diabetic mice could be partly explained by increased intestinal cholesterol absorption, as it has been previously reported in patients and experimental models of diabetes mellitus [55–57].

In addition to mediating m-RCT, HDL lipoproteins have other anti-atherosclerotic properties, such as protecting LDL against oxidative modification [27]. Compelling evidence suggests that this atheroprotective function of HDL lipoproteins is also impaired in diabetes mellitus [24,58,59]. Consistently, the protection against LDL oxidation by HDL isolated from Stz-vehicle was poorer than that shown by HDL isolated from Ctrl-vehicle mice. Administration of CORM-2 to diabetic mice failed to improve HDL-mediated protection against LDL oxidation. The antioxidant ability exhibited by HDL has also been partly attributed to the content of PON-1 [60]. HDL-associated PON-1 is low in patients with diabetes mellitus and leads to dysfunctional HDL with impaired antioxidant capacity [24]. In this regard, circulating levels of PON-1 were significantly reduced in both groups of diabetic mice and were not altered by the administration of CORM-2.

In conclusion, the present study shows that a short-term intervention with a high dose of CORM-2 inhibits diabetic neuropathy and oxidative stress but also increases plasma cholesterol levels due to an elevation in the plasma levels of all circulating lipoproteins. Moreover, the administration of CORM-2 also showed an unfavorable effect on the entire m-RCT pathway, and failed at improving the defective antioxidant ability of HDL in our animal model of diabetes mellitus. Insomuch as both m-RCT and antioxidant function of HDL are considered as two of the main antiatherogenic properties of HDL, our data would suggest that, despite improving neuropathy, CORM-2 treatment might not be favorable in protecting against atherosclerotic cardiovascular disease in diabetes mellitus type 1. Therefore, more experiments are needed to test lower doses and more prolonged treatment with CORM-2 to reduce painful diabetic neuropathy without side effects.

## Supporting information

S1 Fig Representative epididymal adipose tissue sections stained with hematoxylin-eosin. (A) Ctrl-vehicle mice. (B) Stz-vehicle mice. (C) Stz-CORM-2 mice. Hematoxylin-eosin staining of epididymal adipose tissue. Scale bar: 20 μm. (D) Averaged adipocyte size in epididymal adipose tissue 5-μm sections. The data are expressed as the mean ± SEM (n = 3–4 mice per group). Differences between the mean values were determined using either a nonparametric Kruskal-Wallis test followed by a Dunn test. Non-overlapping fields for each animal were selected indiscriminately and histopathologically analyzed by a pathologist who was blind to the treatment given. Adipocyte size was measured using NIH ImageJ. Note that adipose tissue from both groups of Stz mice (B and C) showed a significant decrease in adipocyte size compared with Ctrl-vehicle mice (A). * indicates significant differences *vs*. Ctrl-vehicle (*p* < 0.05). CORM-2, (tricarbonyldichlororuthenium(II) dimer; Ctrl, control mice; Stz, streptozotocin treated mice.
(TIF)

S2 Fig **Pearson correlation** between the plasma levels of FFA and (A) epididymal fat weight and (B) plasma levels of triglycerides and (C) non-HDL-cholesterol. Data are expressed as Pearson r. Values considered for correlation analysis were plasma levels of FFA and the epididymal fat weight of all mice (n = 15). A parametric Spearman’s test was used to study the extent of association between parameters. FFA, free fatty acids.
(TIF)

S1 Table Correlation between pain metrics and *Bdkrb1* or *Cybb* gene expression. Data are represented as the Pearson correlation coefficient and r square. The linear correlation between variables was calculated by means of the Pearson correlation test.
(DOCX)

S2 Table Effect of CORM-2 on epididymal adipose mRNA expression levels of molecular determinants of triglyceride mobilization. Data are expressed as the mean ± SEM (n = 3 per group). Differences between the mean values were determined using either a nonparametric Kruskal-Wallis test followed by a Dunn test or parametric one-way ANOVA followed a Student-Newman-Keuls test, as appropriate. CORM-2, (tricarbonyldichlororuthenium(II) dimer; Ctrl, control mice; *Lipe*, hormone-sensitive lipase; *Lpl*, lipoprotein lipase gene; Stz, streptozotocin treated mice.
(DOCX)

S3 Table Effect of treatments on the hepatic gene expression of *Cd36*. Data are expressed as the mean ± SEM (n = 5 per group). A nonparametric Kruskal-Wallis test followed by a Dunn test was used to compare differences between groups. * indicates significant differences *vs*. Ctrl-vehicle (*p* < 0.05). CORM-2, (tricarbonyldichlororuthenium(II) dimer; Ctrl, control mice; Stz, streptozotocin treated mice.
(DOCX)

S4 Table Effect of CORM-2 on the HDL composition and surrogate of HDL size of diabetic mice. Data are expressed as the mean ± SEM. A nonparametric Kruskal-Wallis test followed by a Dunn test was used to compare differences between groups. The surface-to-core ratio was calculated by dividing surface (amphipathic or less apolar) components (ie, protein, phospholipids, and free cholesterol) by core (highly apolar) components (ie, triglycerides and cholesteryl esters). Similarly, the protein-to-cholesterol ratio was calculated by dividing the protein by the cholesterol component of HDL, whereas protein-to-lipid ratio was calculated by dividing the protein by the lipid component of HDL. CORM-2, (tricarbonyldichlororuthenium(II) dimer; Ctrl, control mice; HDL, high-density lipoprotein; Stz, streptozotocin treated mice.
(DOCX)

S5 Table Effect of CORM-2 on hepatic and small intestine mRNA expression levels of molecular determinants of m-RCT. Data are expressed as the mean ± SEM (n = 5 per group). Differences between the mean values were determined using either a nonparametric Kruskal-Wallis test followed by a Dunn test or parametric one-way ANOVA followed a Student-Newman-Keuls test, as appropriate. * indicates significant differences *vs*. Ctrl-vehicle treated mice (*p* < 0.05). CORM-2, (tricarbonyldichlororuthenium(II) dimer; Ctrl, control mice; Stz, streptozotocin treated mice.
(DOCX)
