# Supplementary material for: Administration of CORM-2 inhibits diabetic neuropathy but does not reduce dyslipidemia in diabetic mice
Source: PLoS One. 2018 Oct 4;13(10):e0204841. doi: 10.1371/journal.pone.0204841 (PMC6171880; doi:10.1371/journal.pone.0204841)
Supplement: S1 Table — Data are represented as the Pearson correlation coefficient and r square. The linear correlation between variables was calculated by means of the Pearson correlation test. (DOCX) [file pone.0204841.s003.docx]

**S1 Table. Correlation between pain metrics and *Bdkrb1* or *Cybb* gene expression.**

|  | *Mechanical allodynia* | *Thermal hyperalgesia* | *Thermal allodynia* |
| --- | --- | --- | --- |
| ***Bdkrb1*** |  |  |  |
| **r** | 0.828 | 0.772 | 0.834 |
| **r^2^** | 0.686 | 0.596 | 0.696 |
| ***p <*** | 0.001 | 0.002 | 0.001 |
| ***Cybb*** |  |  |  |
| **r** | 0.799 | 0.829 | 0.890 |
| **r^2^** | 0.638 | 0.687 | 0.793 |
| ***p <*** | 0.003 | 0.002 | 0.000 |

Data are represented as the Pearson correlation coefficient and r square. The linear correlation between variables was calculated by means of the Pearson correlation test.
