# Supplementary material for: Administration of CORM-2 inhibits diabetic neuropathy but does not reduce dyslipidemia in diabetic mice
Source: PLoS One. 2018 Oct 4;13(10):e0204841. doi: 10.1371/journal.pone.0204841 (PMC6171880; doi:10.1371/journal.pone.0204841)
Supplement: S2 Table — Data are expressed as the mean ± SEM (n = 3 per group). Differences between the mean values were determined using either a nonparametric Kruskal-Wallis test followed by a Dunn test or parametric one-way ANOVA followed a Student-Newman-Keuls test, as appropriate. CORM-2, (tricarbonyldichlororuthenium(II) dimer; Ctrl, control mice; Lipe, hormone-sensitive lipase; Lpl, lipoprotein lipase gene; Stz, streptozotocin treated mice. (DOCX) [file pone.0204841.s004.docx]

**S2 Table. Effect of CORM-2 on epididymal adipose mRNA expression levels of molecular determinants of triglyceride mobilization.**

|  | ***Ctrl*** | ***Stz*** | |  |
| --- | --- | --- | --- | --- |
| **Gene targets** | ***vehicle*** | ***vehicle*** | ***CORM-2*** | ***P*** |
| *Lipe* | 1.0 ± 0.0 | 2.2 ± 0.5 | 1.8 ± 0.3 | 0.14 |
| *Lpl* | 1.0 ± 0.1 | 1.3 ± 0.1 | 0.8 ± 0.1 | 0.17 |

Data are expressed as the mean ± SEM (n=3 per group). Differences between the mean values were determined using either a nonparametric Kruskal-Wallis test followed by a Dunn test or parametric one-way ANOVA followed a Student-Newman-Keuls test, as appropriate. CORM-2, (tricarbonyldichlororuthenium(II) dimer; Ctrl, control mice; *Lipe*, hormone-sensitive lipase; *Lpl*, lipoprotein lipase gene; Stz, streptozotocin treated mice.
