# Supplementary material for: Administration of CORM-2 inhibits diabetic neuropathy but does not reduce dyslipidemia in diabetic mice
Source: PLoS One. 2018 Oct 4;13(10):e0204841. doi: 10.1371/journal.pone.0204841 (PMC6171880; doi:10.1371/journal.pone.0204841)
Supplement: S3 Table — Data are expressed as the mean ± SEM (n = 5 per group). A nonparametric Kruskal-Wallis test followed by a Dunn test was used to compare differences between groups. * indicates significant differences vs. Ctrl-vehicle (p < 0.05). CORM-2, (tricarbonyldichlororuthenium(II) dimer; Ctrl, control mice; Stz, streptozotocin treated mice. (DOCX) [file pone.0204841.s005.docx]

**S3 Table. Effect of treatments on the hepatic gene expression of *Cd36*.**

|  | *Ctrl* | *Stz* | |  |
| --- | --- | --- | --- | --- |
|  | *vehicle* | *vehicle* | *CORM-2* | *p* |
| ***Cd36*** | 1.0 ± 0.2 | 4.4 ± 1.1 * | 5.8 ± 1.5 * | < 0.01 |

Data are expressed as the mean ± SEM (n=5 per group). A nonparametric Kruskal-Wallis test followed by a Dunn test was used to compare differences between groups. * indicates significant differences *vs.* Ctrl-vehicle (*p* < 0.05). CORM-2, (tricarbonyldichlororuthenium(II) dimer; Ctrl, control mice; Stz, streptozotocin treated mice.
