# Supplementary material for: Administration of CORM-2 inhibits diabetic neuropathy but does not reduce dyslipidemia in diabetic mice
Source: PLoS One. 2018 Oct 4;13(10):e0204841. doi: 10.1371/journal.pone.0204841 (PMC6171880; doi:10.1371/journal.pone.0204841)
Supplement: S4 Table — Data are expressed as the mean ± SEM. A nonparametric Kruskal-Wallis test followed by a Dunn test was used to compare differences between groups. The surface-to-core ratio was calculated by dividing surface (amphipathic or less apolar) components (ie, protein, phospholipids, and free cholesterol) by core (highly apolar) components (ie, triglycerides and cholesteryl esters). Similarly, the protein-to-cholesterol ratio was calculated by dividing the protein by the cholesterol component of HDL, whereas protein-to-lipid ratio was calculated by dividing the protein by the lipid component of HDL. CORM-2, (tricarbonyldichlororuthenium(II) dimer; Ctrl, control mice; HDL, high-density lipoprotein; Stz, streptozotocin treated mice. (DOCX) [file pone.0204841.s006.docx]

**S4 Table. Effect of CORM-2 on the HDL composition and surrogate of HDL size of diabetic mice.**

|  | *Ctrl* | *Stz* | |  |
| --- | --- | --- | --- | --- |
| **Parameters** | *vehicle* | *vehicle* | *CORM-2* | *P* |
| Free cholesterol [%] | 1.33 ± 0.12 | 1.75 ± 0.49 | 1.13 ± 0.44 | 0.54 |
| Cholesteryl esters [%] | 21.74 ± 1.93 | 20.63 ± 2.36 | 19.63 ± 2.98 | 0.49 |
| Triglycerides [%] | 1.62 ± 0.28 | 1.30 ± 0.35 | 0.94 ± 0.12 | 0.20 |
| Phospholipids [%] | 25.72 ± 0.19 | 25.96 ± 1.38 | 25.38 ± 0.57 | 0.86 |
| Protein [%] | 50.57 ± 0.56 | 52.11 ± 0.87 | 53.03 ± 0.95 | 0.10 |
| Surface-to-core ratio | 3.45 ± 0.43 | 3.17± 0.11 | 3.33 ± 0.08 | 0.23 |
| Protein-to-cholesterol ratio | 2.25 ± 0.23 | 2.30± 0.15 | 2.28 ± 0.10 | 0.58 |
| Protein-to-lipid ratio | 1.01 ± 0.05 | 1.03 ± 0.02 | 1.08 ± 0.04 | 0.98 |

Data are expressed as the mean ± SEM. A nonparametric Kruskal-Wallis test followed by a Dunn test was used to compare differences between groups. The surface-to-core ratio was calculated by dividing surface (amphipathic or less apolar) components (ie, protein, phospholipids, and free cholesterol) by core (highly apolar) components (ie, triglycerides and cholesteryl esters). Similarly, the protein-to-cholesterol ratio was calculated by dividing the protein by the cholesterol component of HDL, whereas protein-to-lipid ratio was calculated by dividing the protein by the lipid component of HDL. CORM-2, (tricarbonyldichlororuthenium(II) dimer; Ctrl, control mice; HDL, high-density lipoprotein; Stz, streptozotocin treated mice.
