# Supplementary material for: Administration of CORM-2 inhibits diabetic neuropathy but does not reduce dyslipidemia in diabetic mice
Source: PLoS One. 2018 Oct 4;13(10):e0204841. doi: 10.1371/journal.pone.0204841 (PMC6171880; doi:10.1371/journal.pone.0204841)
Supplement: S5 Table — Data are expressed as the mean ± SEM (n = 5 per group). Differences between the mean values were determined using either a nonparametric Kruskal-Wallis test followed by a Dunn test or parametric one-way ANOVA followed a Student-Newman-Keuls test, as appropriate. * indicates significant differences vs. Ctrl-vehicle treated mice (p < 0.05). CORM-2, (tricarbonyldichlororuthenium(II) dimer; Ctrl, control mice; Stz, streptozotocin treated mice. (DOCX) [file pone.0204841.s007.docx]

**S5 Table. Effect of CORM-2 on hepatic and small intestine mRNA expression levels of molecular determinants of m-RCT.**

|  | *Ctrl* | *Stz* | |  |
| --- | --- | --- | --- | --- |
| **Gene targets** | *vehicle* | *vehicle* | *CORM-2* | *p* |
| **Liver** |  |  |  |  |
| *Apoa1* | 1.0 ± 0.2 | 0.6 ± 0.2 | 0.7 ± 0.1 | 0.1 |
| *Abca1* | 1.0 ± 0.2 | 0.4 ± 0.2 * | 0.3 ± 0.1 * | < 0.05 |
| *Abcg1* | 1.0 ± 0.2 | 0.5 ± 0.1 | 0.6 ± 0.2 | 0.18 |
| *Abcg5* | 1.0 ± 0.3 | 1.1 ± 0.3 | 1.5 ± 0.4 | 0.61 |
| *Abcg8* | 1.0 ± 0.3 | 1.6 ± 0.3 | 1.4± 0.5 | 0.72 |
| *Scarb1* | 1.0 ± 0.1 | 1.2 ± 0.1 | 1.3 ± 0.2 | 0.62 |
| *Cyp7a1* | 1.0 ± 0.1 | 1.6 ± 0.4 | 1.5 ± 0.4 | 0.67 |
| *Abcb11* | 1.0 ± 0.5 | 0.4 ± 0.1 | 0.4 ± 0.1 | 0.49 |
| **Small intestine** |  |  |  |  |
| *Abcg5* | 1.0 ± 0.5 | 0.4 ± 0.1 | 0.4 ± 0.1 | 0.34 |
| *Abcg8* | 1.0 ± 0.2 | 0.7 ± 0.2 | 0.8 ± 0.2 | 0.15 |
| *Npc1l1* | 1.0 ± 0.5 | 0.6 ± 0.2 | 0.6 ± 0.2 | 0.78 |

Data are expressed as the mean ± SEM (n=5 per group). Differences between the mean values were determined using either a nonparametric Kruskal-Wallis test followed by a Dunn test or parametric one-way ANOVA followed a Student-Newman-Keuls test, as appropriate. * indicates significant differences *vs.* Ctrl-vehicle treated mice (*p* < 0.05). CORM-2, (tricarbonyldichlororuthenium(II) dimer; Ctrl, control mice; Stz, streptozotocin treated mice.
